# Effect of *Daesiho-tang* on obesity with non-alcoholic fatty liver disease: a study protocol for a randomised, double-blind, placebo-controlled pilot trial

**DOI:** 10.1186/s13063-020-4068-y

**Published:** 2020-01-31

**Authors:** Kyungsun Han, Ojin Kwon, Hyo-ju Park, So-Young Jung, Changsop Yang, Chang-Gue Son

**Affiliations:** 1 Clinical Medicine Division, Korea Institute of Oriental Medicine, Daejeon, Republic of Korea; 2 Liver-Immune Research Center, Dunsan Hospital of Daejeon University, Daejeon, Republic of Korea

**Keywords:** Obesity, Non-alcoholic fatty liver disease, Herbal medicine, *Daesiho-tang*, *Dai-saiko-to*, *Dachaihu-tang*

## Abstract

**Background:**

The high prevalence of obesity and non-alcoholic fatty acid disease has become an important public health problem. *Daesiho-tang* (DST) is an herbal medicine widely used to treat obesity, metabolic syndrome and liver diseases. This pilot study will assess the feasibility of using DST in obese patients with a non-alcoholic fatty liver disease (NAFLD) prior to undertaking a full-scale clinical trial.

**Methods/design:**

This is a study protocol for a randomised, double-blind, parallel-group, stratified, placebo-controlled pilot trial. We will recruit a total of 60 participants with NAFLD who have a body mass index ≥ 25 kg/m^2^. They will take either DST or placebo (3 g, three times daily) for 12 weeks with a 4-week follow-up period. The effects of DST will be evaluated by the mean change in body weight as the primary measurement and other secondary parameters (body composition, anthropometric measurements, blood tests, hepatic fat quantification through transient elastography and a physical symptoms questionnaire). Faecal samples will be collected before and after the intervention for a gut microbial analysis.

**Discussion:**

In anticipation of conducting further large-scale trials, in this study we will explore the effect of DST on weight loss and obesity-related markers, along with NAFLD-related clinical parameters, in obese patients with NAFLD. Furthermore, it will provide insight into the DST pharmacological mechanism of action through a gut microbiome analysis.

**Trial registration:**

Korean Clinical Trial Registry, KCT0003554. Registered on 25 February 2019.

## Introduction

Approximately 39% of the world’s population was overweight or obese in 2015, and this figure will reach about 58% in 2030 [1, 2]. Obesity itself is considered a serious disease because it increases the risk of developing multiple diseases, such as diabetes mellitus, cardiovascular diseases, osteoarthritis and several cancers [3, 4]. Obesity is also the main cause of non-alcoholic fatty liver disease (NAFLD). NAFLD has the characteristic of hepatic fat accumulation in patients who do not use alcohol and may lead to insulin resistance, coronary heart disease, diabetes and serious liver diseases [5–7]. Authors of a retrospective cohort study reported that people with fatty liver have a three times increased risk of de novo metabolic syndrome [8]. Therefore, drugs for obesity and NAFLD are needed, but no definitive treatment exists. In general, NAFLD treatment is managed according to obesity, type 2 diabetes mellitus and hyperlipidaemia [9].

*Daesiho-tang* (DST; *Daichaihu-tang*, 大柴胡湯 in Chinese) is an herbal medicine widely used to treat obesity, metabolic syndrome and liver diseases in East Asian countries. DST consists of eight medical herbs, and the main active components are paeoniflorin, naringin, baicalin, baicalein, sennoside A, rhein, saikosaponin A and emodon [10]. Many animal studies have confirmed the anti-obesity, anti-diabetic and anti-hypertensive effects of DST [11–14]. Hussain et al. reported that DST ameliorates body weight gain in mice fed a high-fat diet by regulating leptin and adiponectin gene expression in adipose tissue [11]. Authors of in vivo and in vitro studies have reported that DST improves lipid metabolism by lowering hepatic triglyceride biosynthesis and by increasing low-density lipoprotein receptor gene expression in hepatocytes [15, 16]. However, no clinical data for DST are available regarding its use for obesity or NAFLD.

This pilot clinical trial is designed to verify the feasibility of DST for later large-scale clinical trials. This study will explore the effect of DST for modulating weight and correcting NAFLD-related parameters and will generate information on a suitable design to conduct further large-scale trials. Additionally, changes in the gut microbial community will be analysed using a sequence-based metagenomic analysis to provide insight into the pharmacological mechanisms of DST in gut microbiota.

## Methods/design

### Study aim and objectives

The aim of this trial is to pilot study procedures and assess feasibility prior to undertaking a full-scale clinical trial on DST to control body weight in obese patients with NAFLD. The primary objective is to assess the feasibility of DST in weight loss compared with placebo in obese patients with NAFLD. The secondary objective is to determine the efficacy of DST in metabolic disease-related parameters, such as waist and hip circumference, body composition, blood analysis, transient elastography analysis, NAFLD-related physical discomfort and obesity-related quality of life. As an exploratory outcome, changes in the gut microbial community will be analysed to evaluate the effect of DST on the gut microbiota.

### Study design

**Fig. 1:**
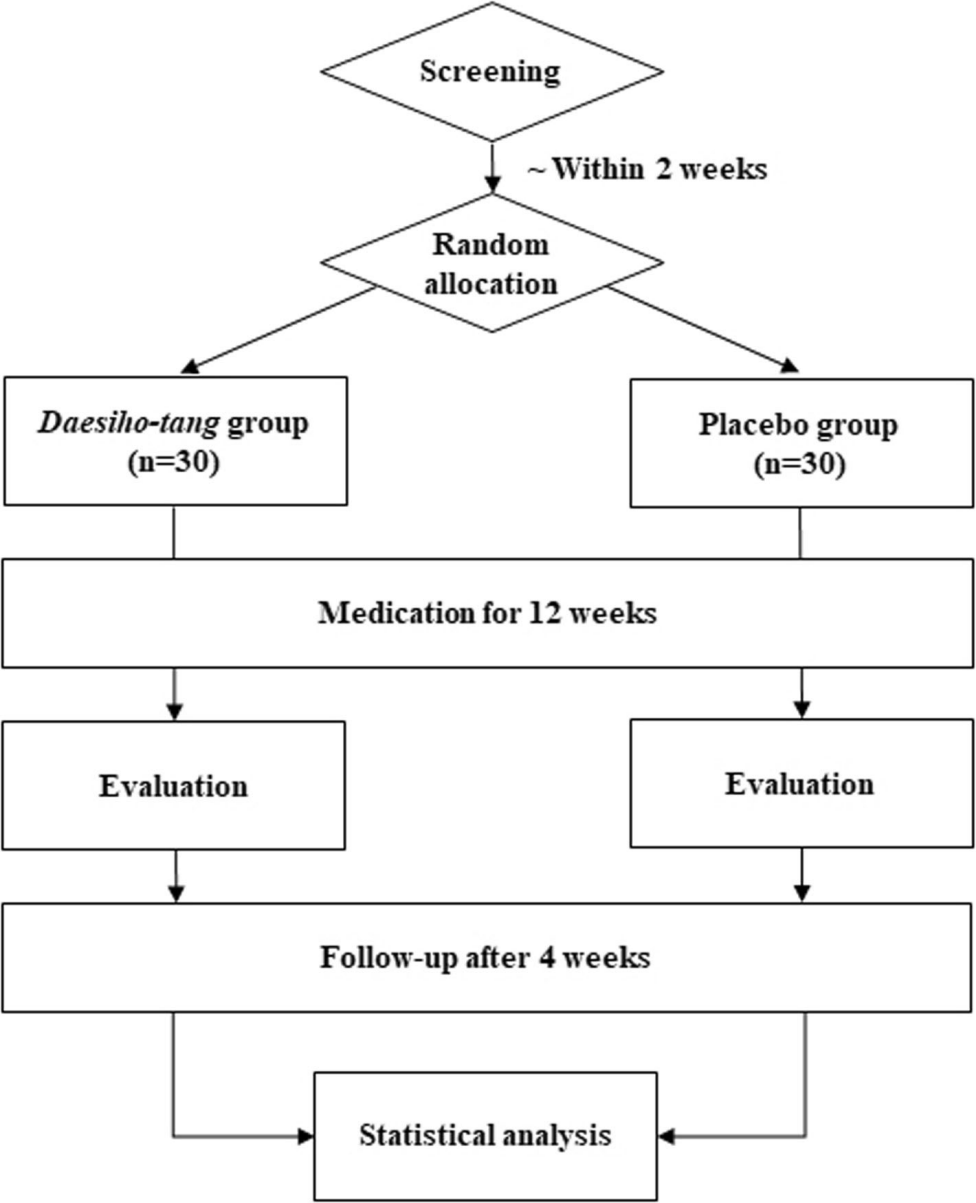
Study flowchart

**Fig. 2:**
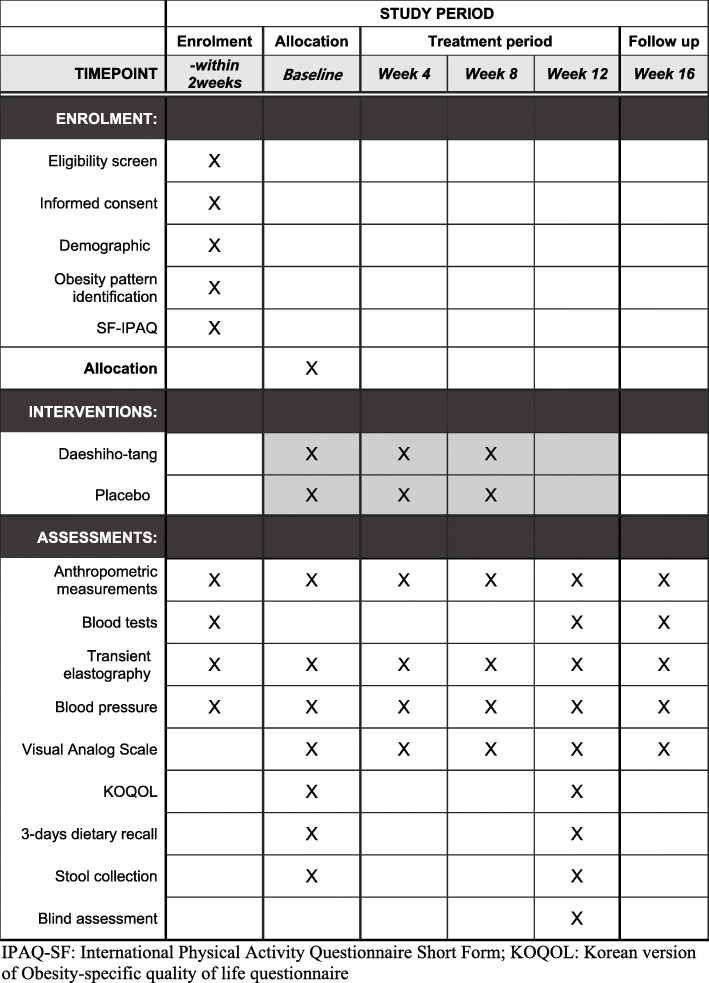
Schedule of enrolment, interventions and assessments

This study is a randomised, double-blind, parallel-group, stratified (by gender), placebo-controlled pilot clinical trial. It will be conducted at Dunsan Korean Medicine Hospital, Daejeon University, Daejeon, Republic of Korea. A total of 60 participants will be recruited by local advertisements and the hospital bulletin board. All participants will be asked to sign the latest version of the informed consent form approved by the local institutional review board (IRB). When subjects voluntarily decide to participate, they will undergo screening, and specialists in Korean medicine will confirm their eligibility based on the inclusion and exclusion criteria. If a subject has been qualified to participate, they will be invited for a baseline assessment within 2 weeks. At the second visit, subjects will be randomly allocated to either the DST group or the placebo group in a 1:1 allocation ratio. Participants will take either DST or the placebo drug for 12 weeks. Participants will visit once monthly for evaluation while taking the test drugs. A follow-up assessment will be performed 4 weeks after the final drug administration. The study flowchart is presented in Fig. 1, and a detailed schedule of enrolment, interventions and assessments is given in Fig. 2.

### Participants

#### Inclusion criteria

1. Adults over 19 years to under 65 years of age
2. Obese subjects with body mass index ≥ 25 kg/m^2^
3. Subjects with non-alcoholic fatty liver findings on transient elastography: controlled attenuation parameter (CAP) score > 268 dB/m
4. Those who have voluntarily signed a written consent form approved by the IRB after sufficient explanation of this study

#### Exclusion criteria

1. Subjects who have had weight changes > 10% of their previous weight within the last 6 months
2. Subjects who are already on a diet or undertaking heavy exercise for weight control purposes
3. Subjects who have undergone surgical procedures for weight control
4. Patients with endocrine disease that may affect body weight, such as hypothyroidism, Cushing’s syndrome, or those with thyroid-stimulating hormone concentration < 0.1 uIU/ml or > 10.0 uIU/ml
5. Patients with evidence of chronic hepatitis, such as hepatitis B, hepatitis C (HCV), autoimmune hepatitis and metabolic hepatitis (hepatitis B surface antigen [HbsAg] or HCV antibody-positive in screening tests)
6. Subjects with alcohol intake > 210 g per week for men and > 140 g for women during the last year
7. Liver stiffness measurement (LSM) of 12.5 kPa or higher by transient elastography
8. Subjects who have received liver transplants
9. Subjects who use insulin injections to control blood glucose concentration
10. Subjects taking thiazolidinediones and high-dose vitamin E that may affect NAFLD
11. Those who have taken drugs that can be used to treat obesity, antidepressants, contraceptives, oral steroids, amphetamines, phenodiazines, cyproheptadines, female hormones or thyroid hormones within 3 months of screening (short-term use of prednisone or equivalent corticosteroids < 10 mg/day within 7 days, which may not affect body weight, will be allowed [17])
12. Those whose aspartate transaminase or alanine transaminase exceeds five times the normal upper limit (200 IU/L)
13. Kidney dialysis patients or those with creatinine concentration more than twice the normal upper limit
14. Those who are in seriously unstable medical condition, such as cardiovascular disease, respiratory disease, gastrointestinal disease, hepatobiliary disease, metabolic disease, endocrine disease, renal disease or problems in the urinary reproductive system and nervous system
15. Those who have had a diagnosis or have been treated for malignant tumours within the last 5 years
16. Women who are pregnant or lactating, or subjects who do not agree to use effective methods of contraception during the clinical trial (Oral contraceptives are not allowed during the clinical trial.)
17. Subjects with genetic problems, such as galactose intolerance, Lapp lactase deficiency, or glucose-galactose malabsorption
18. Subjects with a history of alcohol abuse or drug abuse within the past year
19. Subjects who have taken other clinical trial drugs within 3 months
20. Subjects who are thought to be inappropriate for this study

### Sample size

This study is a pilot clinical trial to explore the feasibility of DST in obese subjects with NAFLD. No clinical trial has evaluated the efficacy of DST for weight loss and/or NAFLD. Therefore, we calculated sample size based on an opinion from an expert in medical statistics and clinical trials similar to this study that had used herbal supplements for weight loss [18, 19]. According to the previous studies, we assumed the mean and standard deviation of body weight would be 2.2 kg and 2.7, respectively. Thus, the sample size was 30 subjects in each group with a significance level of 5%, power of 80% and drop-out rate of 20%. A total of 60 subjects will be recruited and divided into two groups of 30 subjects each.

### Randomisation and allocation concealment

Subjects will be randomly allocated to either the DST or placebo group in a 1:1 allocation ratio. Among the various factors affecting the development of obesity and NAFLD, gender differences are the most important factor [20–22]. Because gender differences in NAFLD may have a different impact on the progression of complications, such as non-alcoholic steatohepatitis (NASH), liver fibrosis and hepatocellular carcinoma, subjects were stratified according to gender [23]. The gender distribution for each group will be controlled by an independent statistician using the stratified block randomisation method. The statistician will use SAS version 9.4 software (SAS Institute, Cary, NC, USA) to generate a random allocation list and will send the list to the pharmaceutical company for packing. The allocation for each subject will be sealed in an opaque envelope and will not be disclosed until the clinical trial is completely over, unless serious adverse events (SAEs) happen.

### Blinding

A statistician who is not involved in the clinical trial will send the randomisation codes to the pharmaceutical company for packing to maintain blindedness of the assessors and the subjects. The placebo drug was manufactured in a form similar to DST by the same pharmaceutical company. It was confirmed in advance that the taste and flavour were similar (Additional file 1). According to the randomisation sequence, drugs will be packed in the same form and will be delivered to the hospital. During the trial period, the pharmacist will supply the drug sequentially according to the randomisation code. To evaluate whether the blinding is successful, a quick questionnaire will be completed at the end of the study. All subjects were asked the same questions at the end of the intervention: ‘Do you think the test drug you took is a real drug or a placebo? And why do you think so’?

### Intervention

Participants in both groups will take DST or the placebo drugs three times daily (3 g/packet at a time) for 12 weeks. The drugs will be distributed on a monthly basis. Both the DST and placebo drugs will be manufactured by Hanpoong Pharmaceutical (Wanju, Republic of Korea) according to good manufacturing practice guidelines. Both drugs will be prepared in the form of dark brown granules. The constituents of DST are shown in Table 1. The DST granules contain DST soft extract, lactose hydrate and corn starch. The placebo drug contains lactose hydrate, corn starch, caramel colour and ginseng-flavoured powder. Ginseng-flavoured powder is a food ingredient without any medicinal effect that only gives off the flavour of ginseng. To assess adherence to the intervention, the subjects will be told to bring the empty packet of the ingested test drug and the leftover. Subjects who have < 70% drug compliance at the end of the study will be dropped out. The research team will periodically contact subjects by phone or text message to improve compliance and check for safety.

**Table 1:** Constituents contained in a single dose of *Daesiho-tang* soft extract

Constituents	Botanical names	Content (g)
Paeoniae Radix (芍藥)	*Paeonia lactiflora* Pallas	1^a^
Scutellariae Radix (黃芩)	*Scutellaria baicalensis* Georgi	1^b^
Zingiberis Rhizoma Recens (生薑)	*Zingiber officinale* Roscoe	1.67
Ponciri Fructus Immaturus (枳實)	*Poncirus trifoliata* Rafinesque	0.67
Bupleuri Radix (柴胡)	*Bupleurum falcatum* Linné	2
Pinelliae Tuber (半夏)	*Pinellia ternata* Breitenbach	1.33
Rhei Radix et Rhizoma (大黃)	*Rheum officinale* Baillon	0.67
Zizyphi Fructus (大棗)	*Ziziphus jujuba* Miller var. inermis Rehder	1

^a^20 mg as paeoniflorin

^b^100 mg as baicalin

### Permission for use of concomitant drugs

Prohibited drugs include agents that can affect the primary outcome of body weight, such as anti-obesity medications, antidepressants, amphetamines, oral contraceptives, hormone drugs, oral steroids, cyproheptadine and phenothiazine. Agents that can affect NAFLD, such as insulin, thiazolidinedione and high-dose vitamin E (> 800 IU per day) are also prohibited. Lipid-lowering agents, anti-diabetic medication and hepatoprotective agents are not included as prohibited drugs, but subjects should not take them as much as possible unless they were taking it from the screening visit. Drugs that can cause significant changes in the gut microbial community, such as antibiotics and lactic acid bacteria, are not prohibited, but subjects should avoid them as much as possible to minimise the effect on intestinal microorganisms.

### Lifestyle guidelines

The subjects will be told to maintain their usual exercise routine and not to change the amount of exercise or kind of exercise they engage in. The Korean version of the International Physical Activity Questionnaire Short Form will be administered to evaluate physical activity status [24]. Daily intake of a low-calorie 20–25-kcal/kg diet will be recommended to increase the weight loss effect. Food intake status will be investigated using 3-day dietary recall before and after the drug intervention. Can-Pro version 5.0 (a computer-aided nutritional analysis program for professionals; Korean Nutrition Society, Seoul, Korea) will be used for the dietary data analysis. Can-Pro is commonly used in Korea to assess nutrient intake because it has a database that reflects the Korean diet.

### Assessments

#### Anthropometric measurements

Body weight will be assessed at every monthly visit, including the screening visit. Waist circumference and hip circumference will be measured according to the World Health Organisation’s instructions at every monthly visit [25]. Anthropometric measurements will be performed by the same person to minimise errors.

#### Body composition

Body composition, such as fat percentage, fat mass and lean body mass, will be measured at every monthly visit using a body composition analyser (InBody 770; Biospace Ltd., Seoul, Korea). The body mass index will be calculated as weight in kilograms divided by height in meters squared.

#### Non-alcoholic fatty liver assessment

Transient elastography, also called a Fibroscan analysis (Fibroscan 530 Compact; Echosens, Paris, France) will be completed by a trained operator after at least 2 h of fasting. The transient elastography analysis is a non-invasive test to assess liver steatosis and fibrosis. Recent studies have shown that the CAP value is a reliable marker to quantify hepatic steatosis [26–28]. LSM values will also be obtained.

#### NAFLD-related symptoms

Physical symptoms related to NAFLD will be assessed using a visual analogue scale (VAS). The VAS is one of the most widely used measures to evaluate subjective symptoms [29–31]. Participants will be asked to mark their degree of fatigue and abdominal discomfort on a 100-mm horizontal line, where 0 indicates ‘no fatigue/discomfort’ and 100 indicates ‘the worst fatigue/discomfort’. Assessors will record the length between the points marked by the subject.

#### Korean version of Obesity-related Quality of Life (KOQOL) questionnaire

The KOQOL questionnaire was developed to evaluate quality of life in obese patients by reflecting the cultural and linguistic expression of Koreans [32]. The reliability and validity of the KOQOL has been verified (Cronbach’s α, 0.6–0.8) [32]. The KOQOL consists of 15 questions and 6 domains: psychological health, physical health, work-related quality of life, routine life, sex life and diet distress (Additional file 2). The total possible score is 60, and the higher the score, the lower the quality of life.

#### Obesity pattern identification questionnaire

The obesity pattern identification questionnaire was developed by the Korean Institute of Oriental Medicine to categorise obese patients into different constitutions, which is the fundamental basis of Korean traditional medicine [33, 34]. Obese subjects can be classified into six patterns: spleen deficiency, yang deficiency, food accumulation, liver depression, phlegm fluid and blood stasis. The questionnaire will be completed at the screening visit (Additional file 3). The obesity pattern of each subject can be used in a subgroup analysis to evaluate whether treatment effects differ according to the obesity pattern.

#### Blood testing

Blood testing will be done at the screening visit, after 12 weeks of drug intervention and at the follow-up visit (week 16). The blood tests will include aspartate aminotransferase, alanine aminotransferase, alkaline phosphatase, total bilirubin, direct bilirubin, γ-glutamyl transpeptidase, blood urea nitrogen, creatinine, protein, albumin, uric acid, white blood cells, red blood cells, erythrocyte sedimentation rate, haemoglobin, haematocrit and platelets. It will also include glucose, insulin, total cholesterol, low-density lipoprotein cholesterol, high-density lipoprotein cholesterol and high-sensitivity C-reactive protein. Haemoglobin A1c will be examined at the screening and after 12 weeks of drug intervention. Thyroid-stimulating hormone, HbsAg, anti-HCV and urine human chorionic gonadotropin (only women of childbearing age) will be evaluated at the screening visit to determine eligibility.

#### Safety assessment

Blood pressure, heart rate and body temperature will be measured at every visit using an automated device. Participants will be asked if they have any physical discomfort. The frequency of adverse events (AEs) will be presented for the safety assessment. Any AEs will be recorded on the electronic case report form (eCRF) after assessing severity and causality. If an SAE occurs, it will be reported to the IRB as soon as possible. The study physicians will follow up with all participants reporting AEs and will perform medical evaluations to determine the cause of the AE and its possible relationship to the interventions. Additional blood tests may be performed at any time if necessary, and during the study, subjects who meet the exclusion criteria will be dropped out of the study for their safety.

#### Gut microbiome analysis

The subjects will be instructed to collect faecal samples within 24 h before visits in weeks 0 and 12 and freeze them until the visit. A stool-collecting kit and instructions will be provided to collect the faecal samples, which will be stored at − 80 °C for further analysis. The microbiome community will be evaluated by 16S rRNA sequencing analysis. The relative abundance of the bacterial taxa in each sample will be obtained, and significant differences in the bacterial taxa between the DST group and the placebo group will be evaluated.

### Outcome measures

Because this is a pilot trial, the recruitment rate and completion rate will be calculated to determine feasibility of the study design. The primary outcome of this study will be the mean change in body weight from baseline to the end of the 12-week intervention. Secondary outcomes will include body composition and anthropometric measurements. A transient elastography and physical symptoms questionnaire will be completed at every monthly visit.

### Monitoring

The Korea Institute of Oriental Medicine will be responsible for quality control throughout the study. An independent clinical research associate will regularly monitor the overall process to determine if the trial is being performed in accordance with the protocol, standard operating procedures and the guidelines for good clinical practice. Monitoring will ensure that the investigators have been trained properly and that written consent has been obtained using the appropriate procedures. Monitoring will also include source data verification and a check for maintenance and completeness of essential documents and the eCRF.

### Data collection and management

All source documents, including informed consent forms, questionnaires and worksheets, will be collected in compliance with standard operating procedures. The data will be collected via an electronic data capture system through eCRFs using the Medidata RAVE data management system (Medidata Solutions Inc., New York, NY, USA). The principal investigator will ensure anonymization of the subjects and the confidentiality of all information collected during the course of the trial. The final dataset of the clinical trial will be accessible to all authors. The principal investigator will coordinate dissemination of data. The results will be submitted for publication in peer-reviewed journals.

### Statistical analysis

The statistical analysis will be conducted by an independent statistician using SAS version 9.4 software. The significance level will be set to 5% in a two-tailed test. The multiple imputation method will be adopted for missing data. The intention-to-treat (ITT) principle will be applied for the primary analysis. The ITT principle will include subjects who meet the full analysis set criteria, including subjects who had the primary outcome assessed once or more except for the baseline measures and who took the test drug at least once. The per-protocol set will be analysed in a supplementary analysis, which will only include subjects who have completed the study as the supplementary analysis.

The independent *t* test or Wilcoxon rank-sum test will be used for continuous variables, and the chi-square test or Fisher’s exact test will be used for categorical variables. Analysis of covariance will be performed for the primary outcome and the continuous data of the secondary outcomes. The model will include baseline values and gender as covariates and treatment as a fixed effect. Student’s *t* test or Wilcoxon’s signed-rank test will be used to compare data before and after the treatment within each group. Categorical data will be analysed using the chi-square test or Fisher’s exact test. Additionally, repeated measures analysis of variance will be used to compare differences in trends per visit.

The incidence of SAEs and AEs related to the treatment will be analysed. The percentage of subjects who experience one or more side effects during the study will be analysed and presented using a descriptive analysis for each group.

## Discussion

To the best of our knowledge, this is the first clinical trial focusing on the therapeutic effects of DST on obesity in patients with NAFLD. Over the years, many anti-obesity drugs have been developed, but only a few have survived in the market because of a significant number of side effects [35]. To this day, central nervous system-targeted agents are widely used for appetite regulation and energy homeostasis, but they are grossly underused due to safety concerns and high cost. Therefore, a new paradigm is needed to succeed in developing anti-obesity drugs. Drugs that target pathways regulating blood glucose and lipid metabolism have shown potential for managing weight and a non-alcoholic fatty liver [35]. Multi-target therapy can be a potential strategy for treating metabolic diseases, with complex pathogenesis through herbal multi-component preparation [36]. Many studies have searched for effective herbal medicines for treating obesity and NAFLD [37, 38]. Among the many candidates, DST has shown potential to treat obesity and NAFLD in many preclinical studies [11–14].

In traditional Korean medicine, pathophysiologically grouping the overall analysis of clinical data to determine the nature and cause of disease in a patient is called ‘pattern identification’ [39]. Diagnosing the pattern helps to choose a suitable herbal medicine for patients. Although DST is widely used for all liver and metabolic diseases apart from the pattern, it is known to have better effect when prescribed in the appropriate patterns. Major function of DST in traditional medicine is to lower excess heat in the liver and stomach by regulating *qi* [40]. From a clinical point of view, DST can be the best prescription for liver depression and food accumulation patterns. However, there has been no research on the relationship between obesity pattern and DST, and the current study will provide a scientific basis for prescription according to the pattern.

The strength of this study is the rigorous design, which is a randomised, double-blind, parallel-group, stratified, placebo-controlled pilot clinical trial. The Standard Protocol Items: Recommendations for Interventional Trials (SPIRIT) 2013 checklist is provided in Additional file 4. The outcomes derived from this study include objective parameters, such as weight, body composition, blood tests and CAP score obtained from a transient elastography, as well as subjective parameters obtained from questionnaires. Generally, NAFLD is a comprehensive term that includes fatty liver, NASH, hepatic cirrhosis and liver cancer [41]. In this study, only subjects with fatty liver and NASH will be included. This pilot study will explore the effect of DST on weight loss and obesity-related markers, along with NAFLD-related clinical parameters, in obese patients with NAFLD. The results of this study will allow opportunities for optimisation of study procedures and to acquire information on a suitable group of people, such as obesity patterns, to conduct further large-scale trials.

Numerous studies have reported that metabolic disorders are cross-linked to changes in the gut microbial community [42–44]. Various factors, including diet, genetic factors, stress and the sleep cycle, can affect the gut microbiota. Modulation of the gut microbiota through herbal medicine can inversely affect human metabolism and adiposity of the host [45, 46]. In this study, faecal samples will be used for the gut microbial analysis. This approach will provide insight into the DST pharmacological mechanism of action through a gut microbiome analysis.

Some limitations of this study should be mentioned. A long-term observation period was not applicable, and an active comparator group, such as anti-obesity drugs, was not included in the study design. Through this study, we will pilot study procedures and assess feasibility for a future full-scale clinical trial.

## Trial status

The most recent version of the protocol is version 1.4 (5 April 2019), and this was approved by the IRB. This trial is currently recruiting participants. Recruitment commenced on 8 March 2019. This study is expected to be complete by December 2020.

## Supplementary information

**Additional file 1.** Daesiho-tang (DST) and placebo drugs for the clinical trial. The test drugs were manufactured in a form similar to the DST and packed in the same packet. It was confirmed in advance that the taste and flavour were similar.

**Additional file 2.** Questionnaires of the Korean Obesity-related Quality of Life (KOQOL) scale.

**Additional file 3.** Obesity pattern identification questionnaire.

**Additional file 4.** Standard Protocol Items: Recommendations for Interventional Trials (SPIRIT) 2013 checklist.

## Data Availability

The final dataset of the clinical trial will be accessible to all authors.

## Abbreviations

AE: Adverse event
CAP: Controlled attenuation parameter
DST: *Daesiho-tang*
eCRF: Electronic case report form
HbsAg: Hepatitis B surface antigen
HCV: Hepatitis C virus
IRB: Institutional review board
ITT: Intention to treat
KOQOL: Korean version of Obesity-related Quality of Life questionnaire
LSM: Liver stiffness measurement
NAFLD: Non-alcoholic fatty liver disease
NASH: Non-alcoholic steatohepatitis
SAE: Serious adverse event
VAS: Visual analogue scale
