# Supplementary figures and images for: Effect of Daesiho-tang on obesity with non-alcoholic fatty liver disease: a study protocol for a randomised, double-blind, placebo-controlled pilot trial
Source: Trials. 2020 Jan 31;21:128. doi: 10.1186/s13063-020-4068-y (PMC6995056; doi:10.1186/s13063-020-4068-y)

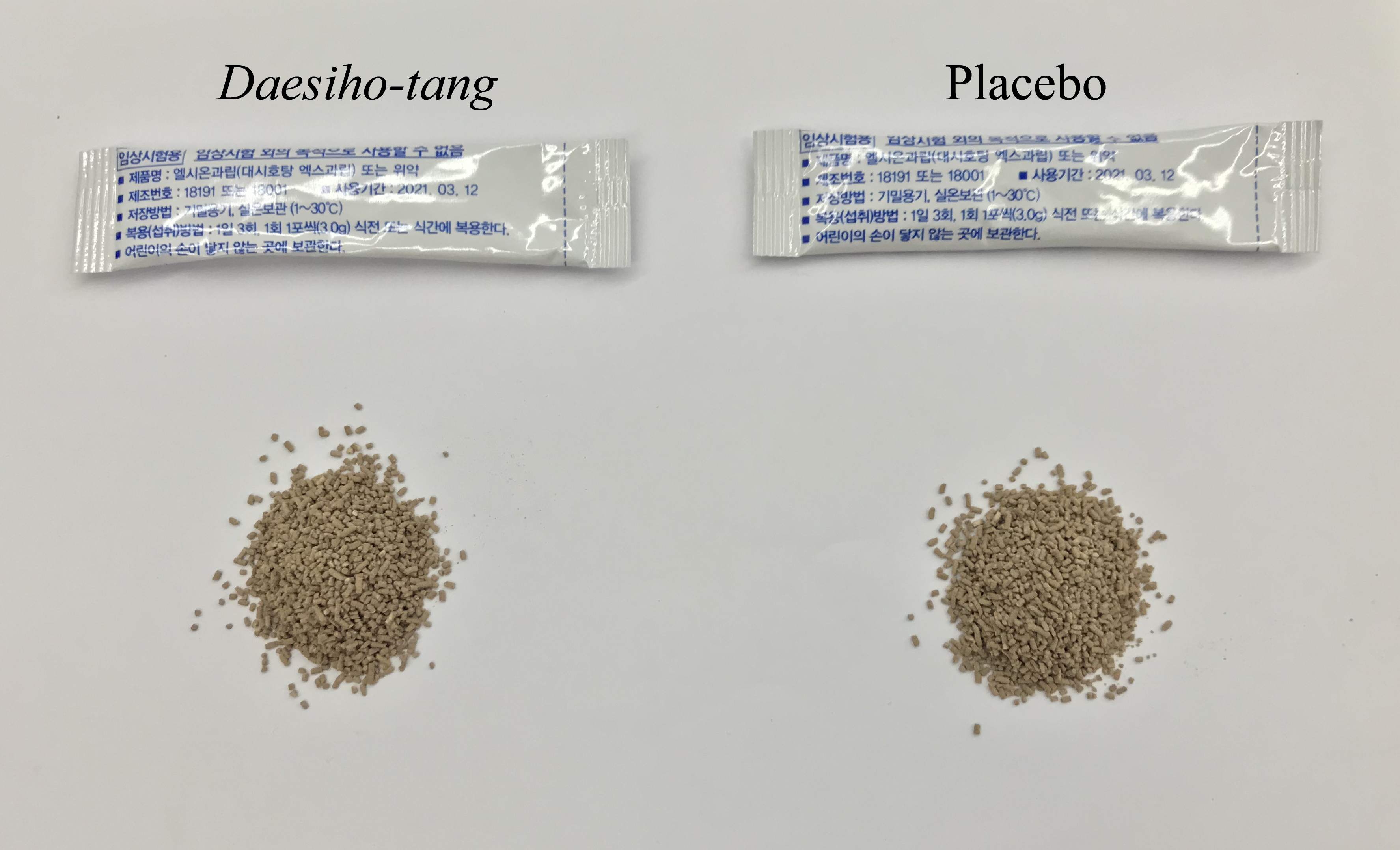

Supplement: Supplementary file 1 — Additional file 1. Daesiho-tang (DST) and placebo drugs for the clinical trial. The test drugs were manufactured in a form similar to the DST and packed in the same packet. It was confirmed in advance that the taste and flavour were similar. [file 13063_2020_4068_MOESM1_ESM.jpg]
