# Supplementary material for: Effect of Daesiho-tang on obesity with non-alcoholic fatty liver disease: a study protocol for a randomised, double-blind, placebo-controlled pilot trial
Source: Trials. 2020 Jan 31;21:128. doi: 10.1186/s13063-020-4068-y (PMC6995056; doi:10.1186/s13063-020-4068-y)
Supplement: Supplementary file 2 — Additional file 2. Questionnaires of the Korean Obesity-related Quality of Life (KOQOL) scale. [file 13063_2020_4068_MOESM2_ESM.docx]

**Additional file 2. Questionnaires for the Korean Obesity-related Quality of Life (KOQOL).** These questions are about how you have been feeling in the last month. Please select the best answers to the questions below.

| Questions | Never  (1) | Sometimes  (2) | Often  (3) | Always  (4) |
| --- | --- | --- | --- | --- |
| Psychological health |  |  |  |  |
| 1. I feel myself to be inferior to others. |  |  |  |  |
| 2. I do not like to meet with other people. |  |  |  |  |
| 3. I feel that I do not look good. |  |  |  |  |
| 4. I feel depressed. |  |  |  |  |
| Physical health |  |  |  |  |
| 5. I am afraid of possible health  complications. |  |  |  |  |
| 6. I have pain in my knee or ankle. |  |  |  |  |
| 7. I experience shortness of breath when I  work out. |  |  |  |  |
| Work-related |  |  |  |  |
| 8. I get lazy and fatigued. |  |  |  |  |
| 9. I am less effective in my work  performance. |  |  |  |  |
| 10. It is hard to work when I crouch. |  |  |  |  |
| Routine life |  |  |  |  |
| 11. I have difficulty using stairways. |  |  |  |  |
| 12. It is hard to find large, well-fitting  clothes. |  |  |  |  |
| Sexual life |  |  |  |  |
| 13. I think I am not sexually attractive. |  |  |  |  |
| 14. I am afraid of having a sexual  relationship. |  |  |  |  |
| Diet distress |  |  |  |  |
| 15. I am concerned about weight gain  whenever I eat. |  |  |  |  |
