# Supplementary material for: Effect of Daesiho-tang on obesity with non-alcoholic fatty liver disease: a study protocol for a randomised, double-blind, placebo-controlled pilot trial
Source: Trials. 2020 Jan 31;21:128. doi: 10.1186/s13063-020-4068-y (PMC6995056; doi:10.1186/s13063-020-4068-y)
Supplement: Supplementary file 3 — Additional file 3. Obesity pattern identification questionnaire. [file 13063_2020_4068_MOESM3_ESM.docx]

**Additional file 3.** **Obesity pattern identification questionnaire**. Please select the best answers to the questions below.

1. Systemic symptoms

| Questions | Severe Never | | | | |
| --- | --- | --- | --- | --- | --- |
| 1. Usually, I feel weak and tired. | 5 | 4 | 3 | 2 | 1 |
| 2. I feel heavy and hate moving around. | 5 | 4 | 3 | 2 | 1 |
| 3. I feel dizzy. | 5 | 4 | 3 | 2 | 1 |
| 4. I feel foggy or have a dull feeling in my head. | 5 | 4 | 3 | 2 | 1 |
| 5. I look pale. | 5 | 4 | 3 | 2 | 1 |
| 6. I get sweaty even though I don’t exercise or get  nervous. I get cold sweats. | 5 | 4 | 3 | 2 | 1 |
| 7. I hate the cold. | 5 | 4 | 3 | 2 | 1 |
| 8. I have cold hands and feet. | 5 | 4 | 3 | 2 | 1 |

2. Sentiment and personality

| Questions | Severe Never | | | | |
| --- | --- | --- | --- | --- | --- |
| 1. I am introverted or sensitive. | 5 | 4 | 3 | 2 | 1 |
| 2. I get easily upset by small things. | 5 | 4 | 3 | 2 | 1 |
| 3. I worry a lot. | 5 | 4 | 3 | 2 | 1 |
| 4. I feel emotionally stifled. | 5 | 4 | 3 | 2 | 1 |
| 5. I am often depressed. | 5 | 4 | 3 | 2 | 1 |
| 6. My pain gets worse when I'm stressed. | 5 | 4 | 3 | 2 | 1 |
| 7. I feel pain when I press my sternum. | 5 | 4 | 3 | 2 | 1 |
| 8. I have a tight feeling in my sides. | 5 | 4 | 3 | 2 | 1 |

3. Digestive functions

| Questions | Severe Never | | | | |
| --- | --- | --- | --- | --- | --- |
| 1. I can’t digest well. | 5 | 4 | 3 | 2 | 1 |
| 2. I can’t digest when I’m stressed. | 5 | 4 | 3 | 2 | 1 |
| 3. I binge or overeat often. | 5 | 4 | 3 | 2 | 1 |
| 4. I tend to eat even when I'm full. | 5 | 4 | 3 | 2 | 1 |
| 5. I have lost my appetite. | 5 | 4 | 3 | 2 | 1 |
| 6. I have small meals. | 5 | 4 | 3 | 2 | 1 |
| 7. I feel heavy in my stomach. | 5 | 4 | 3 | 2 | 1 |
| 8. I burp often. | 5 | 4 | 3 | 2 | 1 |
| 9. I have gas in my bowels. | 5 | 4 | 3 | 2 | 1 |
| 10. I often have an upset stomach. | 5 | 4 | 3 | 2 | 1 |
| 11. I feel like food is stuck in my stomach. | 5 | 4 | 3 | 2 | 1 |
| 12. I often have a stomachache after a meal. | 5 | 4 | 3 | 2 | 1 |
| 13. Sometimes I feel heavy in my stomach and feel  like throwing up. | 5 | 4 | 3 | 2 | 1 |
| 14. I often have soft stool or diarrhoea. | 5 | 4 | 3 | 2 | 1 |
| 15. I have diarrhoea and hear bowel sounds often. | 5 | 4 | 3 | 2 | 1 |
| 16. I usually hear water in my stomach. | 5 | 4 | 3 | 2 | 1 |
| 17. I usually have hard bowel movements. | 5 | 4 | 3 | 2 | 1 |
| 18. I have a problem with urination. | 5 | 4 | 3 | 2 | 1 |

4. Cardiovascular functions

| Questions | Severe Never | | | | |
| --- | --- | --- | --- | --- | --- |
| 1. I often swell all over my body. | 5 | 4 | 3 | 2 | 1 |
| 2. My face or eyes swell often. | 5 | 4 | 3 | 2 | 1 |
| 3. My arms and legs swell often. | 5 | 4 | 3 | 2 | 1 |
| 4. My lower body tends to swell more than my upper  body. | 5 | 4 | 3 | 2 | 1 |
| 5. I often feel stinging pain in my body. | 5 | 4 | 3 | 2 | 1 |
| 6. I have fixed pain in a specific part of my body. | 5 | 4 | 3 | 2 | 1 |
| 7. The pain gets worse at night. | 5 | 4 | 3 | 2 | 1 |
| 8. I have recently been injured. | 5 | 4 | 3 | 2 | 1 |
| 9. Haemorrhagic symptoms appear on my body. | 5 | 4 | 3 | 2 | 1 |
| 10. I bruise often or I can easily see the blood vessels in my skin. | 5 | 4 | 3 | 2 | 1 |
| 11. I have a dark skin colour around my eyes or lips. | 5 | 4 | 3 | 2 | 1 |
| 12. My skin easily becomes cracked. | 5 | 4 | 3 | 2 | 1 |
| 13. I can feel lumps on my skin or stomach. | 5 | 4 | 3 | 2 | 1 |
| 14. I have lumps or a fixed mass somewhere in my body. | 5 | 4 | 3 | 2 | 1 |
| 15. I often have pain in my lower abdomen area,  including menstrual irregularity or menstrual pain  for women. | 5 | 4 | 3 | 2 | 1 |
| 16. I cough frequently and have a lot of sputum. | 5 | 4 | 3 | 2 | 1 |
| 17. It feels like there's something in my throat. | 5 | 4 | 3 | 2 | 1 |
| 18. I have a tight chest and sometimes I feel pain in  my chest. | 5 | 4 | 3 | 2 | 1 |
